# Enzymatic modification of cotton fibre polysaccharides as an enabler of sustainable laundry detergents

**DOI:** 10.1038/s41598-024-73128-x

**Published:** 2024-09-27

**Authors:** Hamish C. L. Yau, James Byard, Lily E. Thompson, Adam K. Malekpour, Timothy Robson, Cassie R. Bakshani, Ieva Lelanaite, William G. T. Willats, Neil J. Lant

**Affiliations:** 1 Procter & Gamble, Newcastle Innovation Centre, Whitley Road, Newcastle upon Tyne, NE12 9BZ UK; 2 School of Natural and Environmental Sciences, Newcastle University, Devonshire Building, Newcastle upon Tyne, NE1 7RU UK; 3 Institute of Microbiology and infection, University of Birmingham, Birmingham, B15 2TT UK

**Keywords:** Biotechnology, Molecular biology

## Abstract

Cotton is the most common natural fibre used in textile manufacture, used alone or with other fibres to create a wide range of fashion clothing and household textiles. Most of these textiles are cleaned using detergents and domestic or commercial washing machines using processes that require many chemicals and large quantities of water and energy. Enzymes can reduce this environmental footprint by enabling effective detergency at reduced temperatures, mostly by directly attacking substrates present in the soils. In the present study, we report the contribution of a cleaning cellulase enzyme based on the family 44 glycoside hydrolase (GH) endo-beta-1,4-glucanase from *Paenibacillus polymyxa*. The action of this enzyme on textile fibres improves laundry detergent performance in several vectors including soil anti-redeposition, dye transfer inhibition and stain removal. Molecular probes are used to study how this enzyme is targeting both amorphous cellulose and xyloglucan on textile fibres and the relationship between textile surface effects and observed performance benefits.

## Introduction

Cotton is an important global textile fibre produced by the genus *Gossypium*, of which ~ 90% of the cotton fibres are cellulose^1^. Cotton fibres are soft, absorbent and breathable, with anisotropic properties that confer a complex morphological structure, making them a popular textile fabric for garment and household textile production^2–5^. Most of the textiles involved are produced by spinning fibres into yarns followed by weaving or knitting the yarns into fabric.

The cellulose microfibril network that accounts for a majority of mature cotton fibres, contains two main regions: (1) crystalline regions which are hydrogen(H)-bonded, organised, non-reactive; and (2) amorphous regions which are less H-bonded, disordered and reactive^6^. Because of these properties, crystalline cellulose content is associated with tensile strength, whilst amorphous regions are associated with hydrophilicity; making these regions susceptible to dye and soil retention^7^.

During textile ageing involving routine wearing and laundering, it is common for textile fibres to be subjected to stresses and strains which contribute to fibre damage and/or surface modifications that expose previously masked cotton epitopes such as amorphous cellulose. This can leave used textiles looking less visually appealing, as well as increasing their propensity to trap dirt and soil, making them appear dingy and fatigued^4,8^. Soil adhesion involves interactions between the soil and fibre chemistry, but it is also influenced by the complex 3D structure of textiles, including pores within the fibre, between fibres in the yarn, and between yarns in the fabric. Gaining an increased understanding of this fibre chemistry could open up new approaches to improve cleaning using enzymes that target the fibres, rather than just the soil components like many established enzyme classes such as protease, amylase, lipase, mannanase and pectate lyase.

Previous studies suggest cleaning of cotton textiles can be improved by “polishing” the surface of textile fibres using cleaning cellulases^8–13^. The proposed hypothesis is that such cleaning cellulases (i) target newly exposed amorphous cellulose epitopes on the fibre surface to release bound soil, (ii) reduce soil adhesion and redeposition, and (iii) open up pore spaces between fibres in yarns to improve cleanability^8,10^. These effects reduce the opportunity for soil redeposition during washing; as well as increasing textile fibre microporosity, which allow soils and stains to be more easily removed and contribute to improved whiteness and cleaning^8,10,14^. Cleaning cellulases differ from so-called care cellulases which have significant activity towards crystalline cellulose and are thus able to remove pills and fuzz from textiles. This activity of care cellulases on crystalline cellulose is accompanied by fabric integrity risk, as fibres, yarns and textiles can become significantly weakened by the action of these enzymes. This risk results in care cellulases only being used at very low levels. However, as cleaning cellulases do not have significant activity on crystalline cellulose, they can be used at higher levels to deliver a different benefit profile without any risk to fabric integrity.

The target region of these cleaning cellulases, amorphous cellulose, is more susceptible to such enzyme degradation due to its looser organisation^7^. Indeed, such cellulases have been used in detergents since the 1980s, to drive consumer relevant benefits on cotton containing textiles^11,13,15–19^. The importance of cellulases also cover applications for animal feed, pulp and paper industries and have therefore been studied extensively^20–23^.

Importantly, cotton fibres also comprise lesser amounts of non-cellulosic polysaccharides including xyloglucan, xylan, pectins and arabinan that are impacted to varying degrees during cotton textile processing^6,24,25^. Xyloglucan is of particular interest as it forms a tethered network with cellulose microfibrils that coat regions of the cellulose surface and provide structural stability to the extensive cellulose network^26–28^. Therefore, xyloglucan is an important polysaccharide to consider alongside cellulose, in a laundry context where improved cleanability is desired through increasing textile microporosity and reducing adhesive surface soil binding sites^8,9,28^.

Cellulases that are functionally different to classic cleaning cellulases have great potential reapplication for textile cleaning and laundry. Amongst these, a GH44 enzyme from *Paenibacillus polymyxa* (*Pp*XG44) has been reported^29^. This enzyme, a truncate of a larger multi-enzyme assembly, has endo-beta-1,4-glucanase activity towards cellulose but also possesses significant activity on xyloglucan^29–31^. The active site of *Pp*XG44 can tolerate branching substitutions, enabling it to accommodate cellulose (undecorated β-1,4 linked polymer of D-glucose) and certain xyloglucan substituents (β-1,4 linked D-glucan polymer carrying branch substitutions comprising xylose units)^29,32^.

Indeed there has been interest in applying the wild-type *Pp*XG44 in detergents^33^, but this molecule is quite unstable in modern liquid detergents leading to development of stabilized variants through protein engineering^34,35^. Two of these *Pp*XG44 variants have been commercialised by Novonesis A/S as ‘Whitezyme’ and ‘Luminous’. In the present work we evaluate the contribution of Whitezyme to several vectors of detergent performance and use molecular probes (antibodies and carbohydrate binding modules) together with microscopy to dissect and illustrate its mechanism of action.

## Results

### Whitezyme enables broad performance benefits on cotton-containing textiles

We first set out to understand the performance profile of Whitezyme by assessing its impact on soil anti-redeposition (whiteness maintenance), dye transfer inhibition and stain removal when added to a heavy-duty liquid (HDL) laundry detergent (UK Ariel HDL) using a tergotometer, commonly used in the detergent industry to simulate a washing machine.

### Whitezyme minimises whiteness loss caused by soil re-deposition through the wash

For anti-redeposition testing, whiteness index (WI) was measured as the amount of white light reflected by a fabric through the visible light spectrum and defined using a whiteness standard set by the International Commission on Illumination (CIE), as WI(CIE)^36^. Change in textile whiteness index (∆WI) after four wash cycles in the presence of soil was calculated as a measure of whiteness loss, thus a less negative ∆WI value defines an improvement in whiteness. Knitted cotton swatches were washed four times in UK Ariel HDL detergent and particulate soil, in the presence or absence of Whitezyme respectively.

**Fig. 1:**
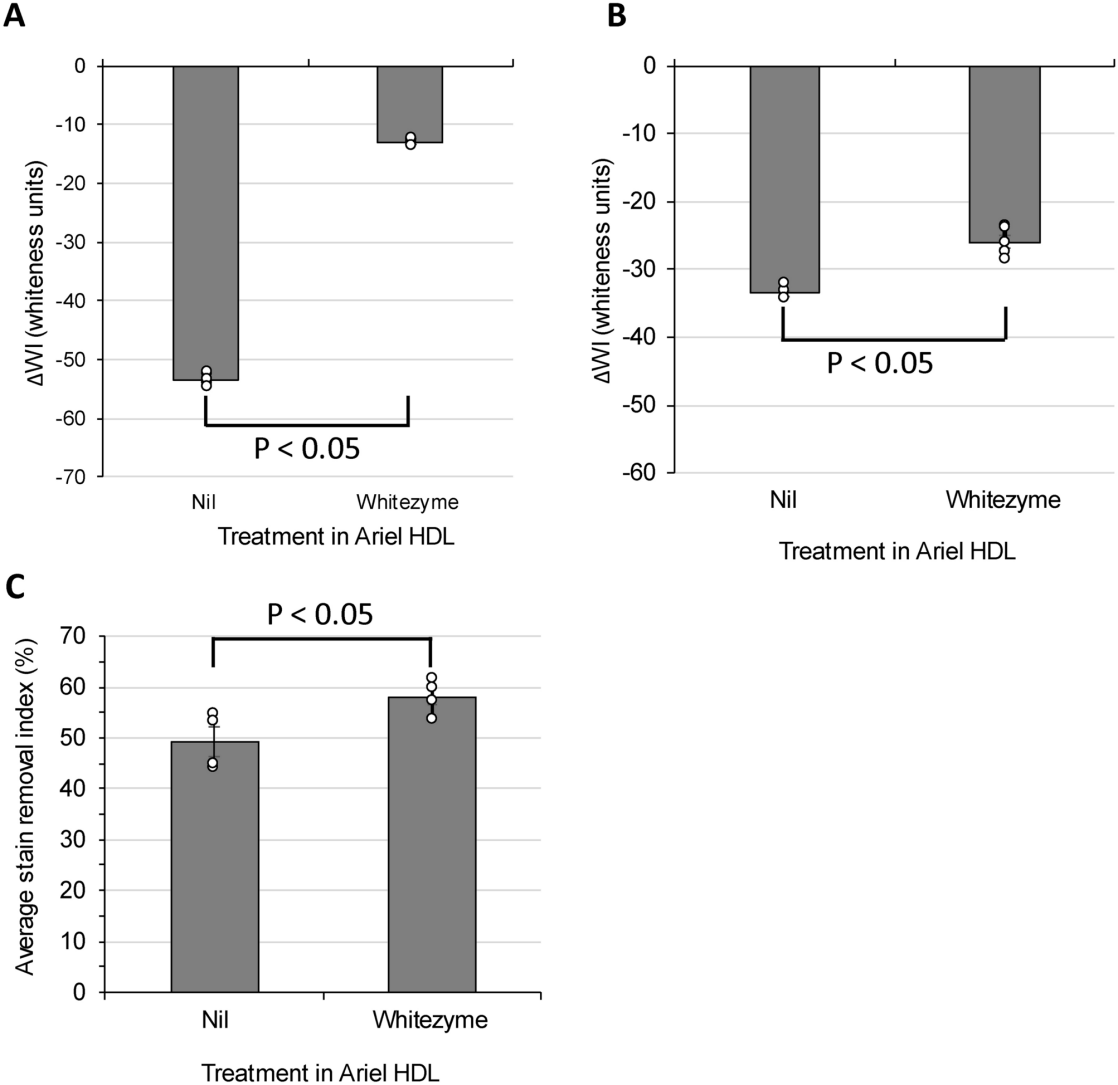
Whitezyme delivers whiteness, soil release, and dye transfer reduction benefits on cotton fabrics - Whitezyme cleaning profile was assessed using cleaning tests ran in automated tergotmeters. (**A**) whiteness benefits were assessed via carbon black removal. Whitezyme added at 0.1 PPM, average WI-CIE ± SEM was plotted (n = 5). (**B**) Dye transfer inhibition (whiteness loss to dye transfer) was assessed by washing Sulfur black dyed fabrics in the presence of clean knitted cotton. Whitezyme added at 0.1 PPM, average WI-CIE ± SEM was plotted (n = 5). (**C**) Removal of chocolate milk drink stains is achieved by washing in 0.5 PPM Whitezyme. Average SRI (%) ± SEM was plotted (n = 4). All assays compared Whitezyme added into UK Ariel HDL *vs* UK Ariel HDL nil Whitezyme. Statistical significance was determined using Student’s t-test; comparisons with a p-value of < 0.050 were judged to be significantly different at 95% confidence level.

Average ∆WI of fabrics washed in Ariel detergent without the cleaning cellulase enzyme (nil) was − 53.4 ± 0.5 whiteness units (Fig. 1A). The resulting textiles were significantly dirtier than fabrics washed in Ariel detergent containing Whitezyme which delivered an average ∆WI of − 12.29 ± 0.2 whiteness units (Fig. 1A). Visual assessments of the fabrics showed very discernible differences in whiteness of cotton fabrics washed in presence and absence of Whitezyme (S. Figure 1A).

### Whitezyme reduces in-wash dye transfer onto treated cotton fabrics

Given that the action of cleaning cellulases involves polishing amorphous cellulose on the surface of cotton fabrics, we next studied possible contributions of Whitezyme to dye transfer inhibition (DTI) given that dyes tend to associate with amorphous cellulose. Dye transfer is an important cause of whiteness and contrast loss in laundry, as dyes applied to fabric are not completely washfast resulting in low levels of dye bleeding, especially in the first few washes of a new item. A wide variety of dyes are used to colour textiles, and these are usually classified based on the mechanism of delivery to the fibre, e.g., Reactive, Direct, Sulfur, Basic, Acid, and Vat. Pigments are also used, especially in printed colour. While many dyes desorb and transfer in water-soluble forms, others including pigments, Sulfur and Vat (e.g. indigo) dyes are released from fabrics as small insoluble particles^37,38^. We hence reapplied the WI assessment described above to measure the level of dye uptake on clean white cotton tracers, from fabrics dyed with Sulfur Black 1, in the presence and absence of Whitezyme. Measuring the ∆WI of tracer fabrics before and after washing, to assess degree of dye transfer, we observed a significantly reduced level of Sulfur Black 1 dye transfer onto clean cotton fabrics washed in detergent containing Whitezyme (− 25.95 ± 1.0 whiteness units) compared to cotton washed in nil Whitezyme detergent (− 33.52 ± 0.4 whiteness units) (Fig. 1B); confirming the qualitative assessment that Whitezyme does indeed confer DTI benefits as part of its cleaning profile (S. Figure 1B).

### Whitezyme confers stain removal benefits against problematic stains containing adhesive cellulosic thickeners

We also evaluated stain removal benefits of Whitezyme against food soils that contain cellulosic rheology modifiers, using chocolate milk stains. Carboxymethyl cellulose (CMC), also known as cellulose gum, is a common food and drink stabiliser that is well known to be adhesive to cotton fabrics^39^. While CMC is formulated as an anti-redeposition polymer in powdered detergents, it is not commonly used in liquid detergents, and little is known about the impact of CMC (in the form of cellulose gum ingredient in foods) on the cleanability of such food soils.

This performance vector is measured in terms of a Stain Removal Index (SRI) which describes the relationship between the appearance of a stain before and after washing. Stain removal is defined as a percentage increase in SRI when calculating delta of post-wash versus pre-wash stains^40^. The average SRI delivered by Ariel HDL without Whitezyme (49.3 ± 2.8%) was statistically lower than the average SRI delivered by the same cleaning detergent system containing Whitezyme (58.1 ± 1.8%) (Fig. 1C). We therefore conclude that Whitezyme also delivers discernible stain removal benefits against cellulose gum-containing stains as part of its performance profile.

### Whitezyme in detergent modifies amorphous cellulose and xyloglucan on the fabric surface

Next, we sought to understand the mechanism of action responsible for the cleaning profile observed in Fig. 1, by testing the hypothesis that Whitezyme-driven cleaning benefits are due to its dual activity against amorphous cellulose and xyloglucan present on the fabric surface^29^.

To assess this, carbohydrate binding module (CBM) families that recognise amorphous cellulose and xyloglucan chains were identified. CBM44a from *Clostridium cellulolyticum* has previously been characterised and reported to recognise amorphous cellulose (HEC) and xyloglucan epitopes^41^. We used a GFP-tagged version of CBM44a (NZYtech) to visualise the presentation of these target substrates on the surface of cotton fabrics preconditioned (washed four cycles in UK Ariel HDL) with 0.5 PPM Whitezyme—a level at which we observe various consumer relevant benefits (Fig. 1).

**Fig. 2:**
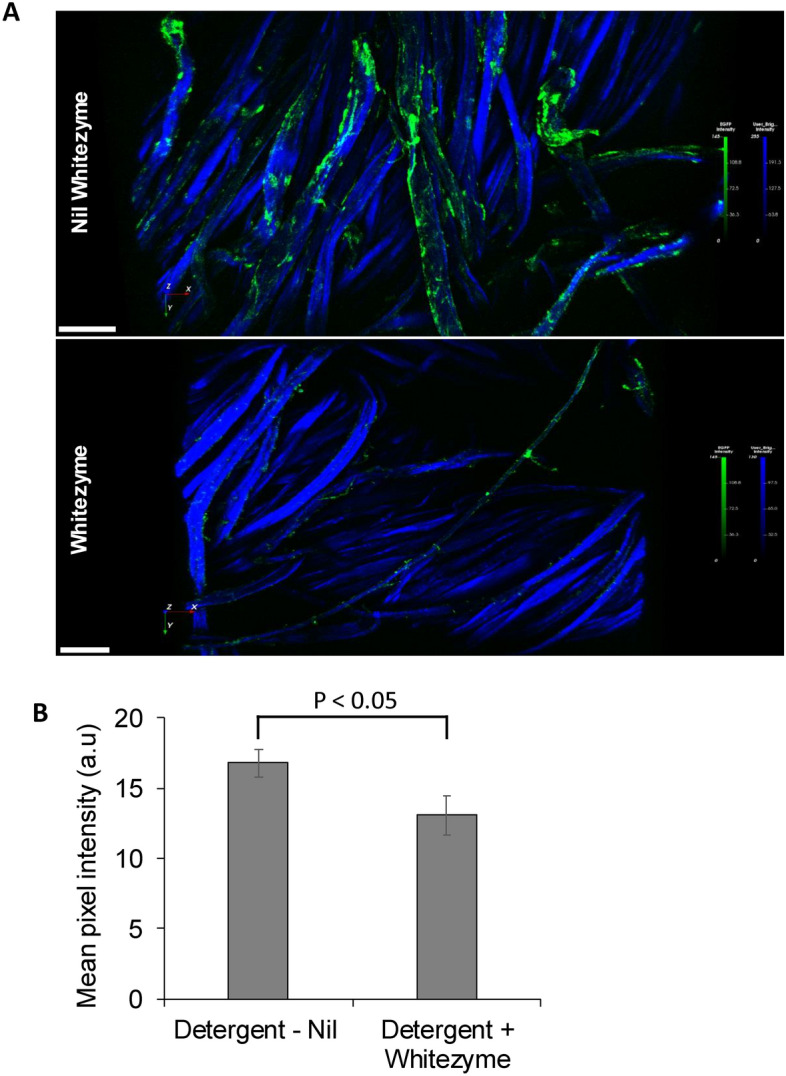
Whitezyme activity in detergent drives a reduction in amorphous cellulose and xyloglucan detected on the fabric surface (**A**) Whitezyme treatment reduces CBM44a-GFP signal on fabric surface. CBM44a recognizes amorphous cellulose and xyloglucan. Representative images from 2 independent imaging experiments displayed. Scale bars = 50 μm (**B**) Quantification of fluorescence intensity. Mean pixel intensity ± range plotted. Statistical significance was determined using Student’s t-test; comparisons with a p-value of < 0.05 were judged to be significantly different at 95% confidence level.

Confocal fluorescence laser scanning microscopy of the washed fabrics revealed that there is a qualitative reduction in amorphous cellulose and xyloglucan on the cotton fabric surface, when Whitezyme is present in the detergent (versus nil Whitezyme detergent) (Fig. 2A). There was a 30.27% reduction in CBM44a-GFP binding intensity, when comparing probe binding on nil Whitezyme *vs* Whitezyme treated fabrics (Fig. 2B, Supplementary data table 4). This confirms that Whitezyme is indeed active on one or a combination of these substrates on fabrics.

To further dissect the action of Whitezyme, molecular probes selective for amorphous cellulose and xyloglucan respectively, were identified and utilised in the present study. The monoclonal antibodies LM15, LM24 and LM25 were selected for their unique specificities to xyloglucan^42–44^, whilst CBM3a from *Clostridium thermocellum* and CBM28a from *Caldicellulosiruptor bescii* were selected for their contrasting affinities to crystalline and amorphous cellulose, respectively^45–51^*.* GFP tagged versions of CBM3a and CBM28a were used to enable their localisation.

**Fig. 3:**
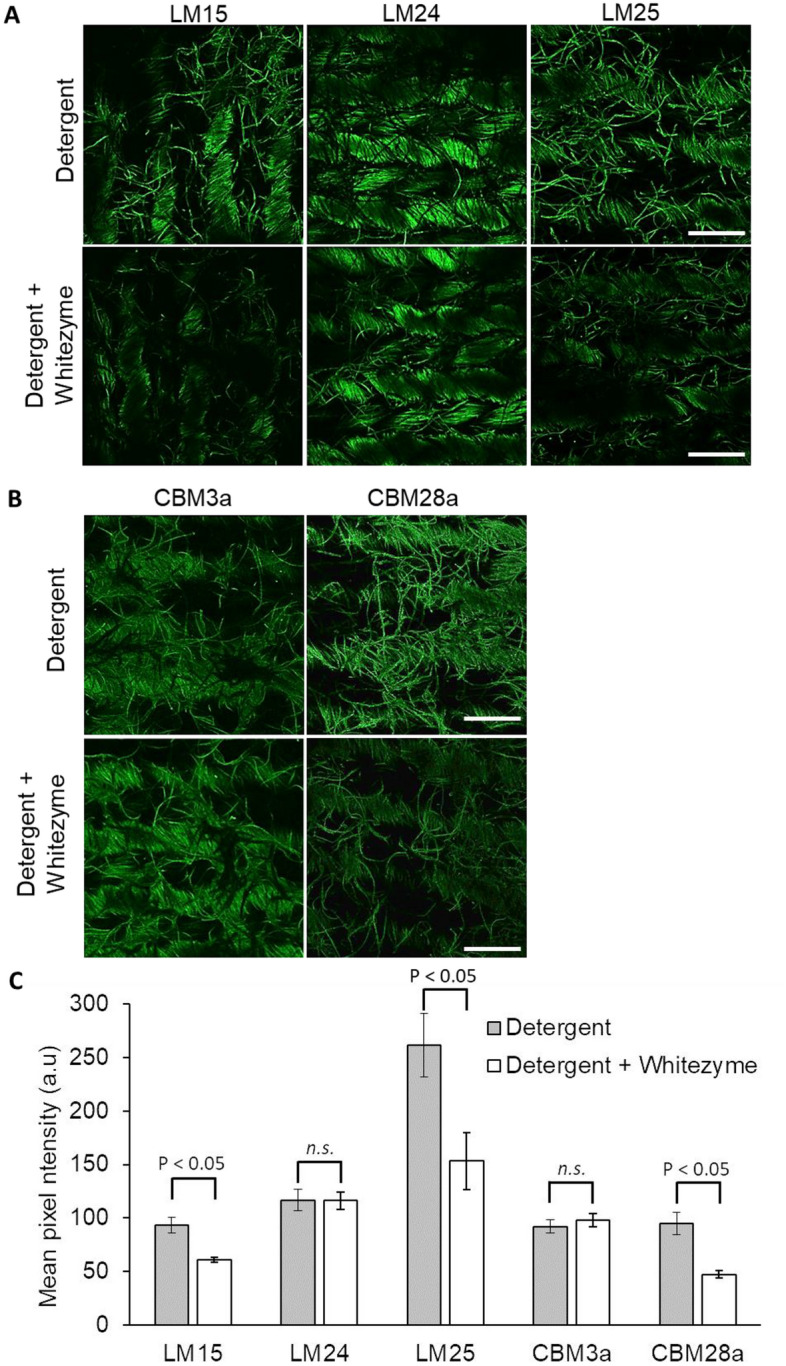
Effect of whitezyme on xyloglucan and cellulose polysaccharides present in cotton fibres (**A**) Whitezyme treatment effect on xyloglucan, scale bars = 750 μm (**B**) whitezyme treatment effect on crystalline and amorphous cellulose, scale bars = 750 μm (**C**) Quantification of fluorescence intensity. Mean pixel intensity ± range plotted. Statistical significance was determined using Student’s t-test; comparisons with a p-value of < 0.05 were judged to be significantly different at 95% confidence level. *N.s, not significant.*

*In situ* fluorescence microscopy revealed that binding of LM15 and LM25 was markedly reduced on Whitezyme treated fabrics vs nil (Fig. 3A); whilst binding of LM24 was comparable on both nil enzyme and Whitezyme treated fabrics (Fig. 3A). LM15 and LM25 bind to xyloglucan with low degrees of substitution, hence we are likely observing a reduction in xyloglucan with low degree of substitution (i.e. XXXG epitopes) processed by Whitezyme^29^. The three-dimensional structure of Whitezyme reveals its inability to tolerate substrates with substitutions beyond three consecutive xylose substituted glucosides. Any further substitutions are reported to bring steric barriers to enzyme–substrate complexation^29^. This likely explains the lack of change to LM24 binding across enzyme-treated and nil-enzyme treated fabrics (Fig. 3A and C), given that LM24 binds to galactosylated xyloglucan oligomers which Whitezyme is unable to accommodate^29^. Overall, we observed statistically significant reductions in LM15 binding (34.6%) and LM25 binding (41.3%) on Whitezyme treated fabrics *vs* Nil Whitezyme treated fabrics (Fig. 3C). In contrast, there was only a negligible 0.42% change in LM24 binding on enzyme treated vs nil-enzyme treated fabrics (Fig. 3C, Supplementary data table 5).

Contrasting changes in presentation of crystalline and amorphous cellulose were observed using CBM3a and CBM28a, respectively (Fig. 3B). Whilst we observed a 50.3% reduction in amorphous cellulose (with CBM28a) following Whitezyme treatment; we observed no reduction to the level of crystalline cellulose (with CBM3a) following Whitezyme treatment (Fig. 3B and C). Interestingly, when comparing the degree of crystalline cellulose binding by CBM3a between 100 to 300 micron fabric depth, there appeared to be slightly higher detection of crystalline cellulose on enzyme treated swatches specifically between 112.5 to 200 micron fabric depth (S. Figure 2, supplementary table 5). It’s likely that given the hierarchical arrangement of amorphous and crystalline cellulose in the primary wall of cotton microfibrils, the surface grazing effect that Whitezyme delivers on amorphous cellulose (and xyloglucan) is subsequently revealing previously embedded crystalline cellulose.

Effects of Whitezyme on other hemicellulosic substrates such as mannans was also studied using a selection of molecular probes which recognise mannan epitopes; LM22, BS-400-4 and CBM27a (S. Figure 3). Under the conditions tested, very little binding signal was observed on nil enzyme treated fabrics and there was no statistically significant increase to mannan accessibility after Whitezyme treatment (S. Figure 3b). This is surprising given that mannans are known to be present in cotton fibres^52,53^. It’s possible that the imaging conditions used in the current study were unable to resolve mannans or that mannans on the detergent treated swatches for the current study are not bound by the respective molecular probes, at the conditions tested. The data also suggests that if mannans are present, the action of Whitezyme against surface exposed amorphous cellulose and xyloglucan, are not sufficient to subsequently expose mannans and elicit an increase in binding intensity (S. Figure 3).

## Discussion

The progressive decay of fabrics, with consequent loss of ‘newness’ that occurs after repeated washing and wearing is an important reason for fashion consumers discarding clothing. Incomplete cleaning, soil redeposition and dye transfer are important mechanisms of decay^54^, suggesting that targeting these areas could have significant environmental benefits by extending the useful lifetime of clothing and driving more sustainable fashion consumption. While many cleaning technologies have large environmental footprints, e.g. through petrochemical origin or poor biodegradability, enzymes have strong environmental credentials as they are biobased, biodegradable, have low manufacturing carbon footprint and are highly efficient requiring little formulation space.

Sustainable alternative technologies to clean clothes are hence of huge interest to society; enabling the solving of tough consumer cleaning problems globally, whilst driving a reduction in scope 3 emissions associated with the laundry process by enabling washing in cold water. Using biodegradable enzymes that are stable in detergent to deliver cleaning in shorter wash cycles, at lower temperatures are one of the strategies being implemented to improve the sustainability of laundry.

The parent molecule of Whitezyme is a GH44 enzyme with dual activity against xyloglucan and amorphous cellulose^29^. It has been hypothesised that such an activity profile would allow an enzyme to polish the surface of worn cotton textile fibres and drive the removal of dirt and soil attached to exposed cellulosic regions, therefore delivering associated cleaning and whiteness benefits^8,9^. To leverage such a benefit profile for more sustainable fashion, the molecule needed to be engineered. Improving stability of this wild-type molecule has been discussed previously^29^, whilst specifically engineering variants for detergent compatibility has also been achieved^34,35^.

**Fig. 4:**
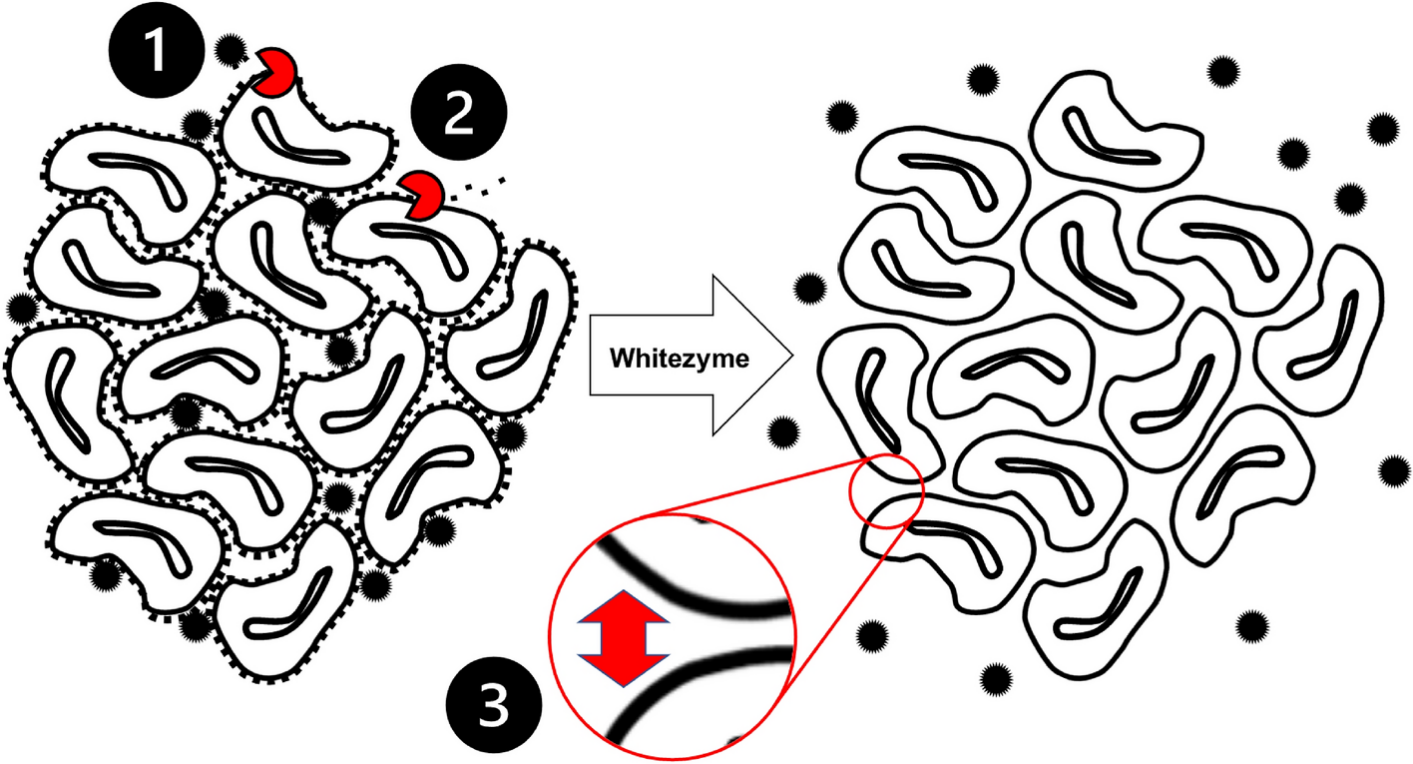
Whitezyme mechanism of action schematic - Illustration summarising the mechanism of action of Whitezyme to drive consumer relevant cleaning benefits. In-figure numbers correspond to the following mechanisms (1) Liberation of soil particles by attacking the amorphous cellulose point of attachment; (2) Reduction of soil redeposition and soil adhesion by removing soil binding sites by polishing the amorphous cellulose on fiber surfaces; (3) Opening up the pores between fibers thus improving the cleanability of the yarn interior by the detergent chemistry (by the same mechanism as 2).

In the present work we have tested one such variant, Whitezyme, and confirmed it is active on both cellulose and xyloglucan (S. Figure 4). We show that the addition of Whitezyme into detergent does indeed deliver a broad consumer-relevant benefit profile, including improved whiteness, dye transfer inhibition and stain removal benefits on cotton textiles (Fig. 1). These observations are consistent with the conceptual model of Calvimontes *et al*., hypothesising that enzymes with endo-β-1,4 glucanase activity (which includes Whitezyme) act by opening the pores between fibres in yarns to improve cleanability of fabrics and align the yarn surface profiles which reduces the surface area for soil pick-up^8^. Together these mechanisms likely contribute to the improved clean appearance of the cotton fabrics observed in the present work.

Interestingly, no statistically significant stain removal benefits were observed when testing other commercial detergent cleaning cellulases with endo-β-1,4-glucanase activity (S. Figure 1C, Supplementary data table 3). This supports the hypothesis that Whitezyme has a unique activity profile versus other cleaning cellulases that drives its superior performance. This further emphasises the relevance of understanding the performance profile of this enzyme, as there could be a huge step-change in cleaning delivered by inclusion of such an enzyme into cleaning detergents.

We have used fluorescence imaging to illustrate reductions in the binding of probes such as CBM44a (recognises xyloglucan and amorphous cellulose), LM15, LM25 (xyloglucan) and CBM28a (amorphous cellulose) on Whitezyme treated fabrics versus nil enzyme (Figs. 2 and 3). Intriguingly, despite the reduced detection of these polysaccharides on the fabric surface, we only observed slight changes in crystalline cellulose bound by CBM3a nearer the surface of the Whitezyme treated fabric (S. Figure 2). We did not observe significant alterations to overall crystalline cellulose on the fabrics, at the conditions tested (Fig. 3B and C). The lack of overall impact on crystalline cellulose would suggest little risk to fabric tensile strength by Whitezyme. This makes Whitezyme an ideal cleaning enzyme as it delivers a good cleaning profile with little risk to fabric integrity.

Interestingly the observed stain removal benefits of Whitezyme, against chocolate milk drink-stained fabrics, was not observed when washing with current commercial detergent cellulases enzymes such as Biotouch FLX (AB enzymes), Celluclean 5.0 T and Endolase 3.0 T (Novonesis A/S) (S. Figure 1C). Comparison of the activity profile of these enzymes suggests the dual activity of Whitezyme against cellulose and xyloglucan is likely contributing to the better stain removal profile of Whitezyme versus current detergent cellulase options which don’t display the dual activity profile (S. Figure 4). This “unusual specificity profile” is consistent with previous characterisation studies on Whitezyme’s parent molecule^29^.

As a result of our study and collation of existing art, variants of *Pp*XG44 such as Whitezyme and Luminous do indeed act as effective detergent enzymes for the modification of specific cotton fibre polysaccharides; delivering additional benefits versus other commercially available cleaning cellulases. The cleaning benefits are likely driven by the following mechanisms: i) decreasing overall yarn surface and liberating soil particles attached to amorphous cellulose, ii) reducing soil binding sites (amorphous cellulose and xyloglucan) on the surface of fabrics to reduce soil redeposition and soil adhesion and iii) increasing microporosity of fabrics thus improving the cleanability of the yarn interior (Fig. 4)^8^. We believe that the substrates processed by Whitezyme are directly linked to consumer-relevant cleaning benefits; hence the much-improved performance in various cleaning vectors versus detergents not containing Whitezyme (Fig. 1, S. Figure 1). The performance of Whitezyme could greatly complement the performance profile of other enzyme classes proposed for use in detergents – i.e. detergent stable lipases for degrading oil and fat or laundry proteases delivering blood stain removal benefits^55,56^.

In summary, Whitezyme is a sustainable technology which could be used in detergents to replace non-biodegradable components currently used to deliver whiteness and cleaning benefits. The present study has summarised the state of the art and provided further detail of Whitezyme’s mechanism of action. The work has illustrated how the consumer cleaning profile of Whitezyme is likely linked to the specific modifications that it delivers on cotton fibre polysaccharides, making cotton surfaces more cleanable and less attractive to dirt. This is further evidence of the value for introducing enzymes as biobased, biodegradable cleaning technologies, as we look to reduce our dependence on non-biodegradable technologies to drive cleaning in shorter and colder washing conditions. Enzymes for cleaning as such, could hence enable more sustainable fashion practices by caring for consumer garments better against the context of more environmentally friendly washing conditions.

## Materials and methods

### Fabric washing

Fabrics were washed in an automated tergotometer (Peerless Systems, Washington, UK) at 35 °C for 40 min (unless otherwise stated), 300 RPM agitation in water measuring 20 US grains per gallon hardness (342 PPM hardness); followed by 2 × 5 min rinse at 15 °C. UK Ariel HDL detergent (batch code: 23600303F0 15:51), was dosed at 2.7 ml/L through the wash, with or without Whitezyme added as indicated.

For whiteness benefit assessment, knitted cotton fabric (5 cm × 5 cm swatches) and clean cotton ballast fabrics totalling 60 g, were washed in the presence of 0.04 g/L Carbon black (Alfa Aeser Chemicals) and UK Ariel HDL detergent with or without 0.1 PPM Whitezyme 2.0L (Novonesis; Batch: JAN00029). This procedure was repeated a further three times and knitted cotton fabric swatches left to dry overnight at ambient room temperature. Colour of fabrics was evaluated before and after washing using the Whiteness index CIE L*a*b* assessment system^36^, measured on a Konica Minolta CM-3610A device, and used to calculate the change in Whiteness index between unwashed (pre-washed) and washed fabrics.

For dye transfer assessment, Sulfur Black I dyed knitted cotton fabrics (Swissatest, product 274) were washed in UK Ariel HDL detergent supplemented with or without 0.1 PPM Whitezyme 2.0L as described above, four times. Dye transfer was assessed using Whiteness index CIE L*a*b* assessment system^36^; measuring delta WI between post and pre-wash (∆WI) UV adjusted values on a Konica Minolta CM-3610A device.

For stain removal assessment, chocolate milk drink stains prepared using Yazoo chocolate milk drink stain (Asda, UK, batch L2303 13:37:45) were washed in UK Ariel HDL detergent supplemented with or without 0.5 PPM Whitezyme 2.0L, as described above for 30 min with a through the wash temperature of 20 °C. Stain removal was calculated by measuring L*a*b* values of washed stain swatches, unwashed stain swatches and unsoiled fabric, using a ColourEye 7000A Solid State Spectrophotometer (X-Rite Europe GmbH)^40^. Delta E* was then calculated by determining the staining intensity for the washed and unwashed stains compared to the unsoiled fabric, using the following equation where the suffix 1 denotes the unsoiled fabric value and the suffix 2 denotes the values for the unwashed or washed stains:

$$\Delta E_{ab}^{ * } = \sqrt {\left( {L_{2}^{ * } - L_{1}^{ * } } \right)^{2} + \left( {a_{2}^{ * } - a_{1}^{ * } } \right)^{2} + \left( {b_{2}^{ * } - b_{1}^{ * } } \right)^{2} }$$

The Stain Removal Index (SRI) is the level of stain removal calculated as a percentage as follows: SRI = 100 x (A – B) / A; Where A represents Delta E* of unwashed fabric-stained region and B represents Delta E* of washed fabric stained region.

### Immunocytochemistry procedures

Molecular probes—Several monoclonal antibodies and CBMs were used in the present work. LM15, LM24, LM25 (KeraFast) recognising different xyloglucan epitopes^42,43^; BS-400-4 (Biosupplies Australia), LM21, LM22 (Kerafast), CBM27a-GFP (NZYtech) to mannans^57,58^; CBM3a-GFP, CBM44-GFP, CBM28a-GFP (NZYtech) recognising cellulose with different degrees of crystallinity^45,46,51^. Anti-rat-FITC and anti-mouse-FITC were purchased from Jackson ImmunoResearch.

Molecular probe imaging of fabrics was achieved as described previously^59^. Briefly, washed 5 cm × 5 cm fabric swatches were cut to 1 cm × 1 cm sections and placed into 6–12 well plates.

GFP conjugated CBM3a, CBM27a, CBM28a and CBM44a (NZYTech) were diluted 1 in 50-fold in 1 × phosphate-buffered saline (PBS) containing 5% (w/v) milk protein (MP-PBS). Fabric samples were incubated with respective solutions at ambient room temperature on a Cole-Parmer Stuart™ orbital rocker plate, set to agitate at 50 RPM, for 1.5 h. Samples were then washed in PBS containing 0.1% (w/v) Tween-20 (PBS-T) once, for 5 min; before a further two times in 1 × PBS only, again for 5 min in darkness. Citifluor AF1 anti-fade mounting solution (Electron Microscopy Sciences) was added to each sample once placed on microscope slides and secured with 1.7 × 2.8 cm adhesive Gene frames (ThermoFisher scientific).

Unconjugated LM15, LM24, LM25, LM21 and LM22 antibodies (Kerafast) were diluted 1 in tenfold MP-PBS^42,44^. BS-400-4 (BioSupplies) was diluted 1 in 50-fold in MP-PBS and used to probe fabrics as described above. Anti-rat-FITC antibody and anti-mouse FITC (Jackson ImmunoResearch) were diluted 1 in 1000-fold MP-PBS and used as secondary antibodies, respectively.

Epi-fluorescence imaging was carried out on a Leica DM6B upright digital research microscope with CTR6 LED electronics box. Image acquisition was carried out using Leica Application Suite X software (3.4.2.18368.1.2) Samples were exposed for 50–200 ms at fabric depths between 10–1000 micron and images captured using, 5 ×—20 × objectives. Confocal imaging was carried out on a Leica Stellaris 5 confocal imaging using the HC PL APO 20x/0.75 CS2 objective. Fluorophores were excited at wavelength 480 nm and emission detected at 507–577 nm. Obtained images were quantified using ImageJ analysis software and processed using Microsoft Excel.

### Molecular biology assays

Activity assays were executed as follows: AZCL-Cellulose (I-AZCEL) and AZCL-Tamarind (I-AZXYG), (both Megazyme) were dispersed in a solution of Ariel UK HDL (2.7 mL/L, 19 US gpg hardness borehole water, pH 7.9) at a concentration of 6 mg/mL. Substrates were incubated with enzymes (0.5 PPM) for 30 min. Samples were centrifuged and absorbance of supernatant measured at 590 nm using Tecan Spark 10 M Multimode reader. An increase in absorbance units denotes enzyme activity.

## Supplementary Information

Supplementary Figures.

Supplementary Tables.

## Data Availability

Raw data included as supplementary data tables 1–6.
