# Supplementary material for: Enzymatic modification of cotton fibre polysaccharides as an enabler of sustainable laundry detergents
Source: Sci Rep. 2024 Sep 27;14:22049. doi: 10.1038/s41598-024-73128-x (PMC11436786; doi:10.1038/s41598-024-73128-x)
Supplement: Supplementary file 1 — Supplementary Figures. [file 41598_2024_73128_MOESM1_ESM.pdf]

Enzymatic modification of cotton fibre polysaccharides as an enabler of sustainable laundry detergents.

Authors and affiliations:

Hamish C. L. Yau[1], James Byard[2], Lily E. Thompson[1], Adam K. Malekpour[1], Timothy Robson[1], Cassie R. Bakshani[2,3], Ieva Lelanaite[2], William G. T. Willats[2] and Neil J. Lant[1]

[1]Procter and Gamble, Newcastle Innovation Centre, Whitley Road, Newcastle upon Tyne, UK, NE12 9BZ.

[2]School of Natural and Environmental Sciences, Newcastle University, Devonshire Building, Newcastle upon Tyne, UK, NE1 7RU.

[3]Institute of Microbiology and infection, University of Birmingham, Birmingham, UK, B15 2TT

Supplementary figures

Supplementary figure legends

Supplementary figure 1 – Representative examples of (A) cotton fabric swatches used for whiteness assessment (B) fabric swatches used for DTI assessment. All swatches shown as either washed with Whitezyme or nil Whitezyme (C) Comparative stain removal index (SRI) of selected market detergent cleaning cellulases vs Whitezyme on chocolate milk drink stains.

Supplementary figure 2 - (A) Fluorescence microscopy images comparing CBM3a binding on nil vs Whitezyme washed fabrics probed at various fabrics depths. (B) Quantification of fluorescence intensities.

Supplementary figure 3 - (A) Representative images obtained by Fluorescence microscopy of nil vs Whitezyme washed fabrics probed with mannan specific antibodies LM22, BS-400-4 and CBM27a. (B) No quantifiable differences in mannan presentation were discerned at the conditions imaged.

Supplementary figure 4 - Assessment of Whitezyme vs detergent cellulase activity against (A) AZCL-cellulose and (B) AZCL-tamarind xyloglucan substrates. Average absorbance of n=4 independent test legs ± SD plotted

Business Use

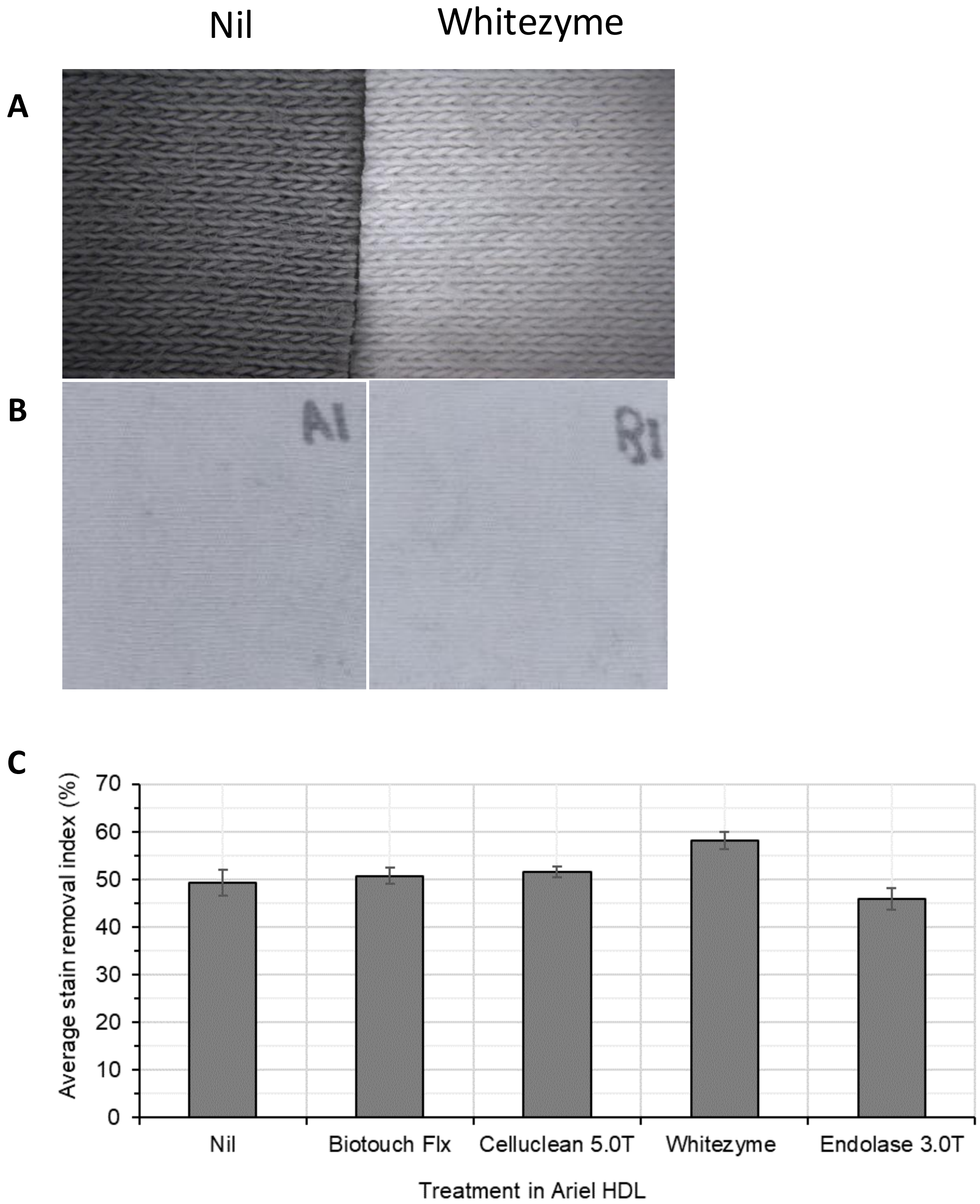

Supplementary figure 1

Representative examples of **(A)** cotton fabric swatches used for whiteness assessment **(B)** fabric swatches used for DTI assessment. All swatches shown as either washed with Whitezyme or nil Whitezyme (**C**) Comparative stain removal index (SRI) of current market cleaning cellulases vs Whitezyme on chocolate milk drink stains.

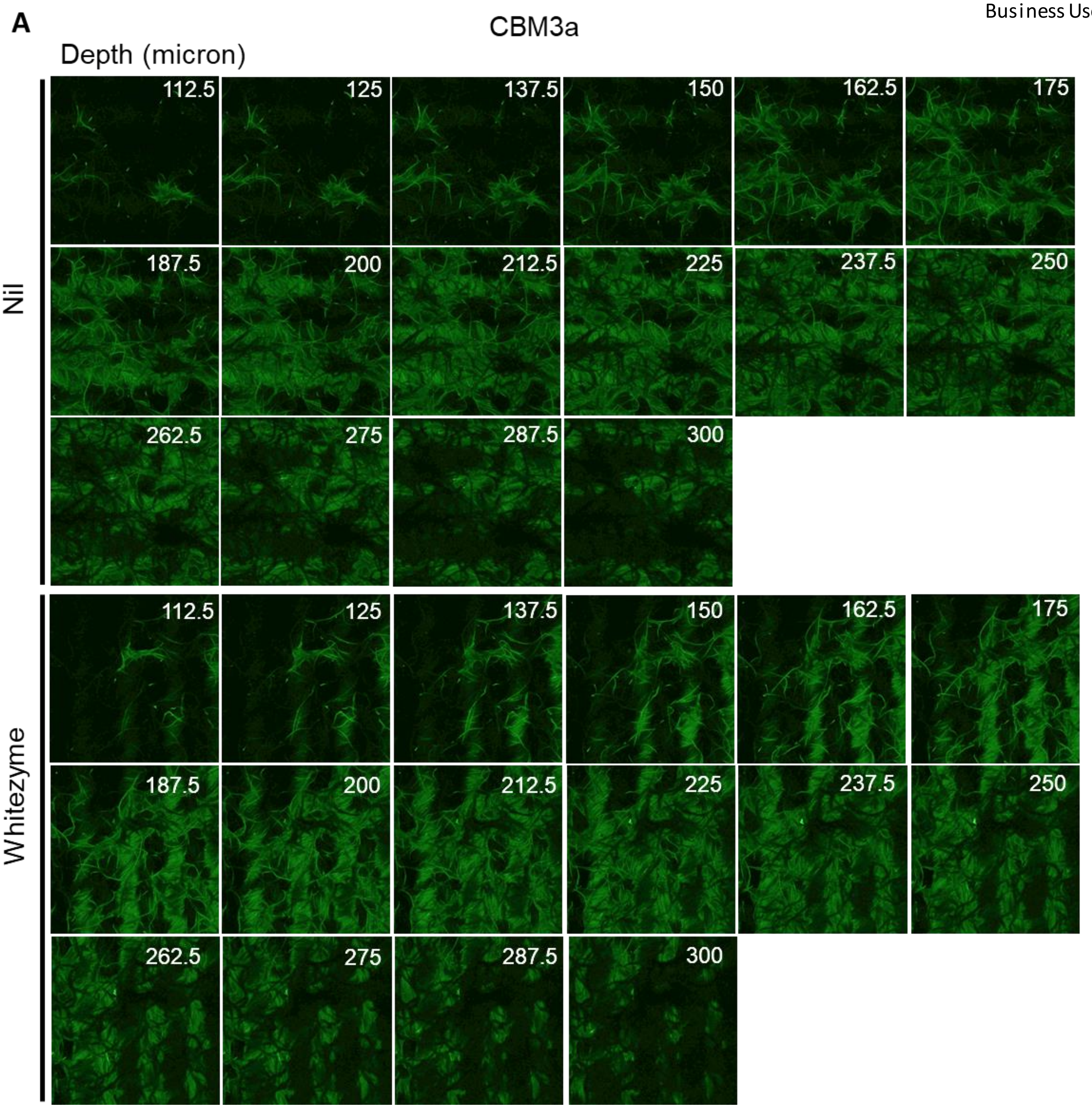

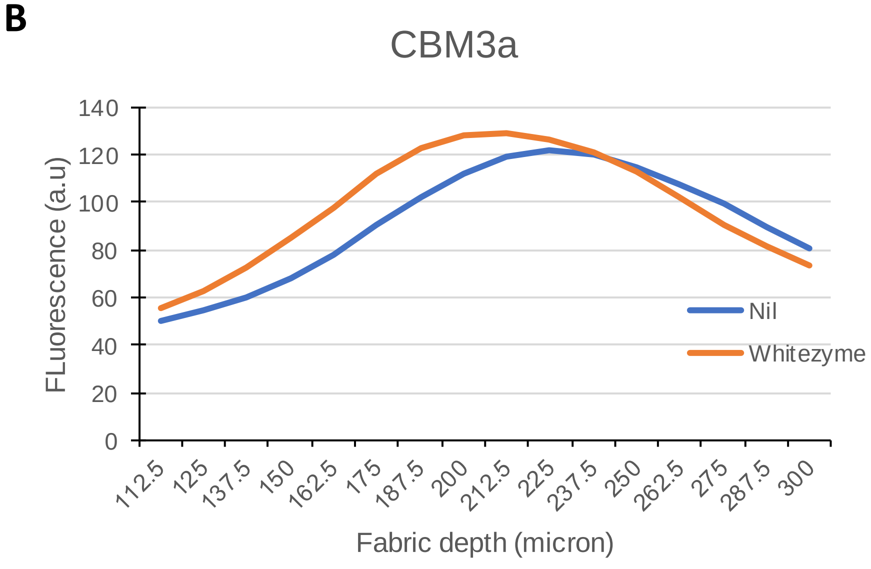

Supplementary figure 2

(A) Fluorescence microscopy images comparing CBM3a binding on nil vs Whitezyme washed fabrics probed at various fabrics depths. **(B)** Quantification of fluorescence intensities.

Business Use

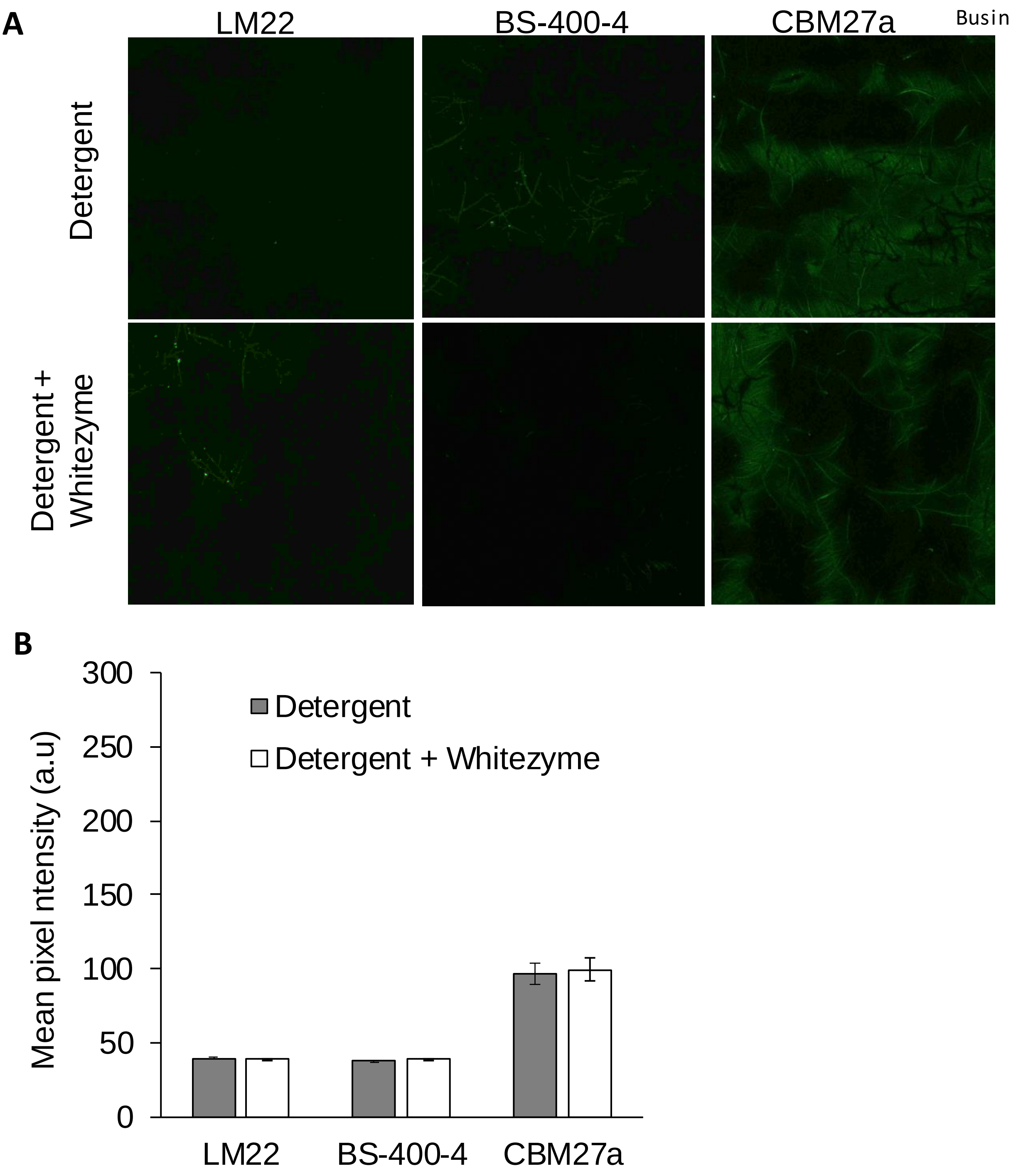

Supplementary figure 3

(A) Representative images obtained by Fluorescence microscopy of nil vs Whitezyme washed fabrics probed with mannan specific antibodies LM22, BS-400-4 and CBM27a. **(B)** No quantificable differences in mannan presentation were discerned at the conditions imaged.

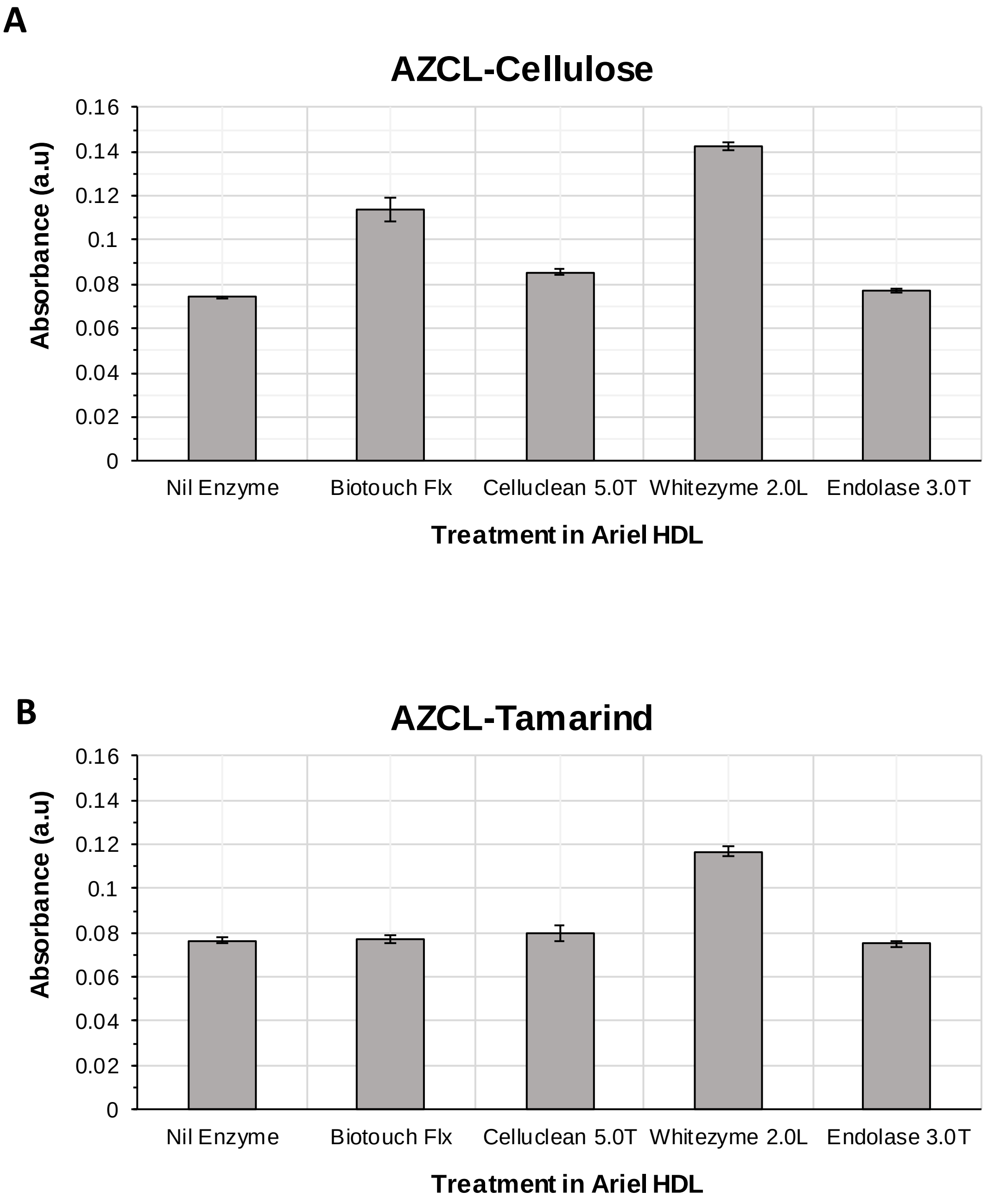

Supplementary figure 4

Assessment of Whitezyme vs detergent cellulase activity against (A) AZCL-cellulose and (B) AZCL-tamarind xyloglucan substrates. Average absorbance of n=4 independent samples ± SD plotted
